# Versorgungsrelevante Daten einer interdisziplinären Sprechstunde für endokrine Orbitopathie

**DOI:** 10.1007/s00347-020-01050-4

**Published:** 2020-02-07

**Authors:** Katharina A. Ponto, Sara v. d. Osten-Sacken, Heike Elflein, Dimitrios Koutsimpelas, Norbert Pfeiffer, George J. Kahaly

**Affiliations:** 1 Augenklinik und Poliklinik, Universitätsmedizin Mainz, Langenbeckstr. 1, 55101 Mainz, Deutschland; 2 I. Medizinische Klinik und Poliklinik, Universitätsmedizin Mainz, Mainz, Deutschland; 3 Hals‑, Nasen‑, Ohrenklinik und Poliklinik, Universitätsmedizin Mainz, Mainz, Deutschland

**Keywords:** Endokrine Orbitopathie, Versorgungsforschung, Interdisziplinäres Orbitazentrum, Morbus Basedow, Epidemiologie, Endocrine orbitopathy, Graves’ disease, Healthcare research, Interdisciplinary orbit center, Epidemiology

## Abstract

**Hintergrund:**

Die endokrine Orbitopathie (EO) geht mit funktionellen Einschränkungen und einer ästhetischen Belastung einher. Ziel der Arbeit war die Untersuchung der Versorgungssituation von Patienten mit EO an einem interdisziplinären Schwerpunktzentrum.

**Material und Methoden:**

Retrospektive Auswertung der interdisziplinären Daten hinsichtlich des klinischen Spektrums, des Versorgungsradius und der Fachrichtung zuweisender Ärzte.

**Ergebnisse:**

Insgesamt wurden die Daten von 431 Patienten mit EO (Frauen: *n* =354, 82 %; Alter [Median]: 40 Jahre; Bereich: 5–79) ausgewertet. 148 (35 %) Patienten wurden vom Hausarzt und 123 (29 %) Patienten vom Augenarzt überwiesen. Eine Optikusneuropathie bestand bei 11 (14,3 %) Männern und bei 21 (5,9 %) Frauen (*p* =0,011). Zusätzlich zur Schilddrüsenerkrankung bestanden mindestens 2 andere Autoimmunerkrankungen bei 8 (10,4 %) Männern und bei 15 (4,3 %) Frauen (*p* =0,079).

Zwei (2,6 %) Männer und 92 (26 %) Frauen nahmen psychotherapeutische Unterstützung in Anspruch (*p* <0,001). Anfahrtswege von 50 km oder mehr nahmen 14 (28 %) Männer und 83 (43 %) Frauen mit EO in Kauf (*p* =0,054). Es bestand eine Assoziation einer Anfahrtsstrecke ≥50 km mit dem Bestehen weiterer Autoimmunerkrankungen (OR: 1,86; 95 %-Konfidenzintervall [KI]: 1,02–3,39; *p* =0,044). Im Trend litten diese Patienten wahrscheinlicher an einer moderat-schweren oder einer sehkraftgefährdenden (1,78, 0,91–3,47; *p* =0,090) EO. Patienten, die einen Anfahrtsweg ≥100 km hatten, waren eher konservativ vorbehandelt (3,78, 1,18–12,05; *p* =0,025).

**Schlussfolgerungen:**

Männer sind im Durchschnitt schwerer von der EO betroffen, haben häufig weitere Autoimmunerkrankungen. Gleichzeitig sind sie der Versorgung schwerer zugänglich. Besonders Patienten mit weiteren Autoimmunerkrankungen nehmen weite Anfahrtsstrecken an ein spezialisiertes Zentrum in Kauf.

## Abstract (en)

**Background:**

Endocrine orbitopathy (EO) encompasses functional and cosmetic limitations. The aim of this study was to assess the health services situation of patients with EO treated at a multidisciplinary specialized center.

**Methods:**

The medical records pertaining to the clinical spectrum, access route, and medical specialty of the referring physician of patients who were treated within a period of 5 years at a tertiary referral orbit center were systematically assessed.

**Results:**

A total of 431 subjects with EO (female *n* =354, 82%; median age 40 years, range 5–79 years) were included in the study. Of the patients 148 (35%) and 123 (29%) were referred by family physicians and ophthalmologists, respectively. A sight-threatening optic nerve neuropathy was present in 11 (14.3%) men and 21 (5.9%) women (*p* =0.011). At least 2 other autoimmune diseases were found in 8 (10.4%) men and in 15 (4.3%) women (*p* =0.079). Psychotherapeutic support was utilized by 2 (2.6%) men and 92 (26%) women (*p* <0.001). An access route of 50 km or more was accepted by 14 (28%) men and 83 (43%) women (*p* =0.054). There was also an association between an access route ≥100 km and a prior medical treatment (odds ratio 3.78, 95% confidence interval 1.18–12.05,* p* =0.025).

**Conclusion:**

Men were more severely affected by EO than women and often had complex autoimmune diseases; however, male patients were less frequently willing to accept long access routes and barely used psychosocial support. Especially patients with further autoimmune diseases travelled long distances to be treated at a specialized center.

Die endokrine Orbitopathie (EO) ist die häufigste extrathyreoidale Manifestation autoimmuner Schilddrüsenerkrankungen und geht in über 90 % mit einem Morbus Basedow einher [22]. Funktionelle Einschränkungen durch Doppelbilder, Hornhautexposition und schlimmstenfalls Kompression des Sehnervs sowie kosmetische Entstellung bei massivem Exophthalmus [3] führen bei einem nicht unerheblichen Anteil an Patienten zu einem Rückzug aus dem sozialen und beruflichen Leben und einer Einschränkung der Lebensqualität [6, 19, 21, 24]. Eine „psychosoziale Morbidität“ der EO wurde anhand eines großen Patientenkollektivs und im Vergleich zur anderen chronischen Autoimmunerkrankungen (Diabetes mellitus Typ 1, entzündliche Darmerkrankungen) nachgewiesen [7].

Die funktionelle und ästhetische Rehabilitation und Verbesserung der Lebensqualität stellen die Leitziele des Mainzer Orbitazentrums dar, das seit 25 Jahren eine interdisziplinäre Sprechstunde für Patienten mit EO anbietet. Basierend auf dieser langjährigen klinischen und wissenschaftlichen Zusammenarbeit, wurden wichtige Fortschritte in der Diagnostik [10, 12, 13, 18, 23] und Behandlung [8, 9, 11, 27] der EO erarbeitet.

Unter Experten besteht Einigkeit über die Notwendigkeit von Orbitazentren für die optimale Versorgung dieser besonders belasteten Patienten. Als Zeichen dieses Konsenses haben sich in Europa verschiedene multidisziplinär arbeitende Zentren etabliert, die im Rahmen der „European Group on Graves’ Orbitopathy“ (EUGOGO) gemeinsame Leitlinien formulieren [1, 2, 20] und Entwicklungen auf dem Gebiet vorantreiben [34]. Zusätzlich zu Expertenkomitees ist die Zusammenarbeit mit Hausärzten, sowie niedergelassenen Endokrinologen, Augenärzten, Nuklearmedizinern und Chirurgen eine wichtige Bedingung für eine patientenzentrierte Versorgung [25, 32]. Bislang fehlen Untersuchungen zur Versorgungssituation von Patienten mit EO in Deutschland. Es existiert lediglich eine Studie zur Schätzung der beruflichen Einschränkungen und der Krankheitskosten der EO [24].

Ziel der Arbeit war die systematische Auswertung von Daten eines Kollektivs von Patienten eines spezialisierten interdisziplinären Orbitazentrums hinsichtlich klinischen Spektrums, psychosozialer Belastung, Anfahrtsstrecke und Fachrichtung zuweisender Ärzte.

## Methoden

Seit 1994 besteht an der Universitätsmedizin Mainz eine wöchentliche interdisziplinäre Orbitasprechstunde: Am gleichen Tag stellen sich Patienten mit EO in der Orbitasprechstunde der Augenklinik und/oder in der Schilddrüsenambulanz vor. Am Nachmittag findet ein zweistündiges interdisziplinäres Board statt, an dem sich Augenarzt und Endokrinologe mit den gemeinsam betreuten Patienten austauschen, die erhobenen Befunde beider Fachabteilungen sowie ggf. zusätzliche Erhebungen anderer beteiligter Disziplinen (z. B. Bildgebung, HNO-Konsil, Histopathologie, psychosomatische Stellungnahmen etc.) diskutieren und darauf basierend das weitere Procedere festlegen. Für die vorliegende Studie wurden die Daten von Patienten retrospektiv ausgewertet, die über einen Zeitraum von 5 Jahren betreut wurden. Einschlusskriterien waren das Bestehen einer gesicherten EO, nachvollziehbare Informationen über Wohnort und/oder Fachrichtung des überweisenden Arztes, eine Betreuung sowohl in der endokrinologischen Ambulanz als auch in der Augenklinik und das Vorhandensein detaillierter Daten über Schwere- und Aktivitätsgrad der EO, Behandlungsstatus der EO, Schilddrüsenerkrankung und -funktion sowie über sonstige Autoimmunerkrankungen und zu Faktoren der Lebensqualität und beruflichen Belastung. Außerdem füllten diese Patienten im Rahmen ihrer Vorstellung in der Sprechstunde für Schilddrüsen- und Autoimmunerkrankungen eine Einverständniserklärung aus, die die Verwendung ihrer pseudonymisierten Daten für Studien und ein Biobanking von Biomaterialien beinhaltete.

Die Einteilung von Aktivität und Schweregrad der EO erfolgte gemäß den Empfehlungen von EUGOGO [2]. Zur Einschätzung der Lebensqualität beantworteten die Patienten einen krankheitsspezifischen Fragebogen, den sog. GO-QOL (Graves’ Orbitopathy – Quality of Life Questionnaire) [33]. Dieser besteht aus jeweils einem Fragenteil zu funktionellen Einschränkungen (Visual Functioning Score) und Einschränkungen der Selbstwahrnehmung (Appearance-Score). Die Antworten werden Punktewerten zugeordnet, aus denen sich 2 Scores berechnen, deren Werte 0 bis 100 annehmen können, wobei höhere Werte einer besseren Lebensqualität entsprechen. Wir definierten keine wesentliche Einschränkung ab einem Wert von mindestens 75 für beide Scores.

Die *statistische* Auswertung erfolgte mit SPSS (Statistical Package for the Social Sciences, Version 25, Chicago, Illinois, USA). Zur explorativen Datenanalyse wurden *p*-Werte bestimmt, die lediglich der Deskription der Daten dienen.

## Ergebnisse

Für die vorliegende Studie wurden die Daten von 431 Patienten mit EO ausgewertet, die die Einschlusskriterien erfüllten. Die Tab. 1 fasst die demografischen und klinischen Daten zusammen. 245 (56,8 %) Patienten kamen aus einem anderen Bundesland als Rheinland-Pfalz, und 5 (1 %) Patienten reisten aus dem Ausland an. Die maximale Wohnortentfernung lag bei 512 km.

**Table 1:** 

**Demografische Daten**	
*Geschlechtsverteilung*	*n (%)*
Männer/Frauen	77 (18)/354 (82)
*Alter bei Diagnose der EO*	*Alter in Jahren*
Median (Bereich)	46 (5–79)
*Dauer der EO bei Einschluss in die Studie*	
≤2 Jahre	(196) 49,4
>2 Jahre	(201) 50,6
*Rauchanamnese*	*n (%)*
Nichtraucher/Raucher	231 (53,6)/200 (46,4)
*Endokrinologische Grunderkrankung*	*n (%)*
Morbus Basedow	407 (94,4)
Autoimmunthyreoiditis Typ Hashimoto	19 (4,4)
Keine	5 (1,2)
*Autoimmunsyndrom*	*n (%)*
Monoglanduläres Autoimmunsyndrom (MGA; eine endokrine Drüse betroffen; z. B. Morbus Basedow/Hashimoto-Thyreoiditis)	335 (77,7)
MGA Typ 2 (zusätzlich eine nichtglanduläre Autoimmunerkrankung; z. B. Morbus Basedow +Autoimmungastritis)	76 (17,6)
Polyglanduläres Autoimmunsyndrom (PGA; mehrere endokrine Drüsen betroffen; z. B. Morbus Basedow +Diabetes mellitus Typ I)	20 (4,6)
*Schilddrüsenfunktion*	*n (%)*
Euthyreose	351 (82,6)
Hypothyreose/Hyperthyreose	9 (2,1)/65 (15,3)
*TSH-Rezeptor-Antikörper*	*n (%)*
Negativ	77 (32,5)
1–10 IU/ml	103 (43,5)
>10 IU/ml	57 (24,1)
**Ophthalmologische Daten**	
*Protrusio bulbi (mm)*, Median (Bereich)	19,5 (8,5–31)
*Doppelbilder*	*n (%)*
Keine Doppelbilder	205 (48,0)
Intermittierend	61 (14,3)
Inkonstant	118 (27,6)
Konstant	43 (10,1)
*Klinische Aktivität der EO*	*n (%)*
Aktiv/inaktiv	173 (40,3)/256 (59,7)
*Schwere der EO*	*n (%)*
Mild	147 (34,6)
Moderat – schwer	256 (60,2)
Sehkraftbeeinträchtigend	22 (5,2)

148 (34,3 %) Patienten wurden vom Allgemeinmediziner und 123 (28,5 %) vom Augenarzt überwiesen. 59 (13,7 %) Patienten kamen von einem Internisten und 41 (9,5 %) ohne Überweisung in die Orbitasprechstunde. Zusammen 60 Patienten (13,9 %) wurden von Nuklearmedizinern (*n* =33) oder Endokrinologen (*n* =27) überwiesen. Bei den vom Nuklearmediziner oder Endokrinologen zugewiesenen Patienten bestand in 4 (12,1 %) bzw. in 3 (11,4 %) Fällen eine Optikusneuropathie (*p* <0,001) und in 4 (12,1 %) bzw. 6 (22,2 %) ein Exophthalmus über 24 mm (*p* =0,009). 24 (72,7 %) bzw. 22 (81,5 %) der vom Nuklearmediziner bzw. Endokrinologen überwiesenen Patienten waren bereits konservativ vorbehandelt (*p* =0,051). Zum Zeitpunkt des Einschlusses in die Studie waren 126 (29,2 %) konservativ und 71 (16,5 %) chirurgisch vorbehandelt. Eine knöcherne Dekompressionsoperation war bei 48 (11,1 %) Patienten, eine Orbitafettgeweberesektion bei 18 (4,2 %) Patienten, eine Schieloperation bei 24 (5,6 %) und Lideingriffe (z. B. Blepharoplastik, Oberlidverlängerung, Unterlidanhebung usw.) bei 31 (7,2 %) Patienten erfolgt (z. T. mehrere Eingriffe pro Patient). Die Untersuchung geschlechtsspezifischer Unterschiede ergab folgende Ergebnisse: Im Vergleich zu Frauen (*n* =354) mit EO lagen bei Männern (*n* =77) häufiger eine Optikusneuropathie (*n* =11; 14,3 % vs. *n* =21; 5,9 %; *p* =0,011), ein Exophthalmus >24 mm (*n* =13; 17,1 % vs. *n* =27; 7,7 %; *p* =0,019) und Doppelbilder (*n* =48; 62,3 % vs. *n* =178; 50,3 %; *p* =0,055) vor. Zusätzlich zur Autoimmunthyreopathie bestanden mindestens 2 zusätzliche Autoimmunerkrankungen bei 8 (10,4 %) Männern und bei 15 (4,3 %) Frauen (*p* =0,079). Bei 11 (14,3 %) der Männer bestand ein polyglanduläres Autoimmunsyndrom (2 endokrine Autoimmunerkrankungen; z. B. zusätzlich zur autoimmunen Schilddrüsenerkrankung ein Diabetes mellitus Typ I oder ein Morbus Addison) vs. bei 9 (2,5 %) der Frauen (*p* <0,001). Ein Nikotinkonsum wurde von 41 (53,2 %) Männern und 159 (44,9 %) Frauen angegeben.

Gleichzeitig nahmen nur 2 (2,6 %) der Männer psychotherapeutische Unterstützung in Anspruch, von den Frauen waren 92 (26 %) in Psychotherapie (*p* <0,001). Daten zum Anfahrtsweg <50 km vs. ≥50 km lagen von 50 Männern und 193 Frauen mit EO vor. Nur 14 (28 %) Männer im Vergleich zu 83 (43 %) Frauen mit EO (*p* =0,054) nahmen Anfahrtswege von 50 km oder mehr in Kauf.

Die Tab. 2 fasst die psychosoziale Belastung (Lebensqualität, Inanspruchnahme von Psychotherapie und berufliche Einschränkungen durch die EO) zusammen. In Bezug auf die Untersuchung möglicher Assoziation zwischen der Wohnortentfernung und klinischen sowie soziodemografischen Daten illustriert Tab. 3 die absoluten und relativen Häufigkeiten.

**Table 2:** 

*Funktionelle Einschränkung der Lebensqualität*	
Visual Functioning Score, Median (Bereich)	75 (0–100)
Deutliche Einschränkung vorhanden (Visual Functioning[VF]-Score <75), *n* (%)	202 (47,4)
Keine deutliche Einschränkung (VF-Score ≥75), *n* (%)	224 (52,6)
*Einschränkung der Selbstwahrnehmung*	
Appearance Score, Median (Bereich)	75 (0–100)
Deutliche Einschränkung vorhanden (Appearance[AP]-Score <75), *n* (%)	183 (43,0)
Keine deutliche Einschränkung (AP-Score ≥75), *n* (%)	243 (57,0)
*Arbeitsunfähigkeit aufgrund der EO pro Jahr*	*n (%)*
Überhaupt nicht	205 (48,1)
<1 Monat	61 (14,3)
1 bis 6 Monate	34 (8,0)
>6 Monate	7 (1,6)
Dauerhaft	14 (3,3)
*Erwerbsminderung aufgrund der EO*	*n (%)*
Nie	231 (53,6)
Vorübergehend	70 (16,2)
Dauerhaft	12 (2,8)
*Inanspruchnahme psychotherapeutischer Betreuung*	*n (%)*
Nein	337 (78,9)
Ja	50 (11,7)
Geplant/Gewünscht	19 (4,4)
Nicht mehr	21 (4,9)

**Table 3:** 

		Anfahrtsstrecke zum Orbitazentrum, *n* (%)			
Kovariaten		<50 km	≥50 km	<100 km	≥100 km
Überweisender Arzt	Allgemeinmediziner	57 (39,0)	37 (38,1)	86 (40,8)	8 (25,0)
	Facharzt	89 (61,0)	60 (61,9)	125 (59,2)	24 (75,0)
Klassifizierung der EO	Mild	56 (38,4)	25 (25,8)	75 (35,5)	6 (18,8)
	Moderat bis sehkraftgefährdend	90 (61,6)	72 (74,2)	136 (64,5)	26 (81,3)
Klinische Aktivität der EO	Inaktiv	91 (63,2)	53 (54,6)	129 (61,7)	15 (46,9)
	Aktiv	53 (36,8)	44 (45,4)	80 (38,3)	17 (53,1)
Arbeitsunfähigkeit aufgrund der EO	Nie	76 (52,1)	37 (38,1)	101 (47,9)	12 (37,5)
	Vorübergehend bis dauerhaft	70 (47,9)	60 (61,9)	110 (52,1)	20 (62,5)
Erwerbsminderung aufgrund der EO	Nie	89 (61,0)	53 (54,6)	125 (59,2)	17 (53,1)
	Vorübergehend bis dauerhaft	57 (39,0)	44 (45,4)	86 (40,8)	15 (46,9)
Inanspruchnahme psychologischer Betreuung	Nein	113 (77,4)	72 (74,2)	160 (75,8)	25 (78,1)
	Ja	33 (22,6)	25 (25,8)	51 (24,2)	7 (21,9)
Dauer der EO seit Diagnosestellung	≤2 Jahre	24 (16,8)	10 (10,5)	32 (15,5)	2 (6,5)
	>2 Jahre	119 (83,2)	85 (89,5)	175 (84,5)	29 (93,5)
Autoimmunerkrankungen	Keine	111 (76,0)	35 (24,0)	152 (72,0)	21 (65,6)
	Mehrere	62 (63,9)	35 (36,1)	59 (28,0)	11 (34,4)
Orbitachirurgie	Keine	120 (82,2)	75 (77,3)	172 (81,5)	23 (71,9)
	Voroperiert	26 (17,8)	22 (22,7)	39 (18,5)	9 (28,1)
Konservative Therapie	Unbehandelt	56 (38,4)	25 (25,8)	76 (36,0)	5 (15,6)
	Vorbehandelt	90 (61,6)	72 (74,2)	35 (64,0)	27 (84,4)

Die multivariate Analyse (Tab. 4) ergab eine positive Assoziation des Anfahrtswegs zum Orbitazentrum ≥50 km mit dem Bestehen weiterer Autoimmunerkrankungen (OR: 1,86; 95 %-Konfidenzintervall [KI]: 1,02–3,39; *p* =0,044). Im Trend waren diese Patienten eher konservativ vorbehandelt (1,80; 0,95–3,43; *p* =0,072) und litten an einer moderat-schweren oder einer sehkraftgefährdenden (1,78, 0,91–3,47; *p* =0,090) EO. Patienten, die eine Anfahrt von 100 km oder weiter in Kauf nahmen, waren eher konservativ vorbehandelt (3,78, 1,18–12,05; *p* =0,025).

**Table 4:** 

	<50 km vs. ≥50 km			<100 km vs. ≥100 km		
	Odds-Ratio	95 %‐Konfidenzintervall	*p*-Wert	Odds-Ratio	95 %‐Konfidenzintervall	*p*-Wert
Autoimmunerkrankungen (Keine vs. mehrere)	*1,86*	*1,02–3,39*	*0,044*	1,50	0,62–3,65	0,367
Konservative Therapie (Unbehandelt vs. vorbehandelt)	*1,80*	*0,95–3,43*	*0,072*	*3,78*	*1,18–12,05*	*0,025*
Klassifizierung der EO (Mild vs. moderat bis sehkraftgefährdend)	*1,78*	*0,91–3,47*	*0,090*	2,47	0,81–7,51	0,110
Überweisender Arzt (Allgemeinmediziner vs. Facharzt)	1,10	0,62–1,96	0,738	2,19	0,86–5,56	0,100
Klinische Aktivität der EO (Inaktiv vs. aktiv)	1,25	0,67–2,33	0,493	1,67	0,71–4,07	0,234
Arbeitsunfähigkeit (Nie vs. vorübergehend bis dauerhaft)	1,55	0,71–3,39	0,269	1,10	0,33–3,64	0,873
Erwerbsminderung (Nie vs. vorübergehend bis dauerhaft)	0,82	0,38–1,77	0,620	0,96	0,31–2,93	0,939
Inanspruchnahme psychologischer Betreuung (Nein vs. ja)	1,06	0,54–2,07	0,874	0,63	0,22–1,81	0,392
Dauer der EO (≤2 Jahre vs. >2 Jahre)	1,77	0,75–4,15	0,190	2,93	0,62–3,65	0,367
Orbitachirurgie (Keine vs. voroperiert)	0,86	0,41–1,82	0,694	1,29	0,48–3,44	0,613

## Diskussion

Die vorliegende Arbeit liefert innovative Daten zur Versorgungssituation von Patienten mit EO, die an einem interdisziplinären Schwerpunktzentrum betreut werden. Es erfolgte eine retrospektive Auswertung der detaillierten endokrinologischen, ophthalmologischen, soziodemografischen und laborchemischen Daten von 431 Patienten, die über einen Zeitraum von 5 Jahren in einer interdisziplinären Orbitasprechstunde betreut wurden. Die *Kernergebnisse* der vorliegenden Arbeit waren folgende: Männer sind schwerer von der EO betroffen als Frauen: Es besteht fast 3‑mal häufiger eine Optikusneuropathie, und mehr als doppelt so häufig entwickelt sich eine ausgeprägte Protrusio bulbi über 24 mm. Männer sind jedoch gleichzeitig einer Versorgung schlechter zugänglich: Sie nehmen weniger weite Anfahrtswege zu einem Therapiezentrum in Kauf und nutzen 10-mal seltener psychotherapeutische Unterstützung. Dass sich Männer seltener oder später an spezialisierten Zentren vorstellen, könnte ein Faktor sein, warum bei Männern häufiger ein besonders schwerer Befund der EO festgestellt wird. Eine weitere mögliche Erklärung für das schwerwiegende klinische Bild bei vielen männlichen Patienten mit EO sind exogene Faktoren, wie z. B. ein erhöhter Nikotinkonsum [28]. Es zeigten sich außerdem Hinweise für einen Zusammenhang von weitem Anfahrtsweg mit dem Bestehen weiterer Autoimmunerkrankungen und schwerwiegenderem und bereits vorbehandeltem Befund. Patienten, die eine besonders weite Anfahrt in Kauf nahmen, waren 4‑mal wahrscheinlicher bereits vorbehandelt. In der vorliegenden Arbeit bestand bei 94,4 % ein Morbus Basedow und bei 4,4 % eine Hashimoto-Thyreoiditis. Eine Euthyreose ohne Nachweis schilddrüsenspezifischer Antikörper bestand zum Zeitpunkt der Datenerfassung bei 1,2 %. Dies deckt sich mit einer eigenen Arbeit zur EO bei initial eu- oder hypothyreoten Patienten: Von insgesamt 461 Patienten des Mainzer Orbitazentrums bestand bei 4,3 % (*n* =20) eine Eu- oder Hypothyreose und bei 95,7 % (*n* =441) ein Morbus Basedow [17]. In einer Arbeit eines anderen deutschen Orbitazentrums waren von insgesamt 182 Patienten mit EO 78,6 % (*n* =143) primär hyperthyreot, 15,4 % (*n* =28) primär euthyreot und 6,0 % (*n* =11) primär hypothyreot [4]. Ein Großteil der Patienten wurde vom Hausarzt oder Augenarzt überwiesen. Patienten mit weiter Anreise kamen häufig auf Anraten eines Endokrinologen oder Nuklearmediziners. Zu dieser Patientengruppe gehörte ein hoher Anteil an Patienten mit Optikusneuropathie, mit gravierendem Exophthalmus sowie bereits vorbehandelten Patienten.

Bei ca. 60 % bestanden ein ausgeprägterer Exophthalmus und/oder einschränkende Doppelbilder. Eine sehkraftgefährdende EO durch Optikusneuropathie bestand bei etwa 5 %. Diese Verteilung deckt sich mit eigenen Daten [22] sowie mit epidemiologischen Daten zur EO [15, 16, 30]. Aus eigenen Vorarbeiten geht hervor, dass die EO nicht selten mit weiteren Autoimmunerkrankungen einhergeht [26]: In einer systematischen Arbeit werteten wir die Daten von 1310 Patienten mit autoimmunen Schilddrüsenerkrankungen aus. Bei 59 % bestand ein Morbus Basedow und bei 41 % eine Hashimoto-Thyreoiditis. Von diesen Patienten bestand bei 13,4 % ein polyglanduläres Autoimmunsyndrom, bei dem zusätzlich zur Schilddrüsenerkrankung eine weitere autoimmune Drüsenerkrankung vorliegt. Ein Diabetes mellitus Typ I war mit 9,8 % die häufigste weitere endokrinologische Autoimmunerkrankung, gefolgt von einem Morbus Addison in 1,9 %. Von den nichtglandulären Autoimmunerkrankungen wurden am häufigsten eine Zöliakie (8,5 %) und eine Autoimmungastritis (4,6 %) nachgewiesen. Es bestand eine positive Assoziation des Bestehens einer begleitenden EO mit einer Zöliakie und einer Autoimmungastritis (Odds-Ratios 3,4 und 4,0). Gerade diese Patienten, aber auch solche ohne Morbus Basedow [17] profitieren von der Expertise des Schwerpunktzentrums auf diesem Gebiet, u. a., weil eine ausführliche Immundiagnostik einschließlich der Messung funktioneller Autoantikörper den klinischen Befund hier sinnvoll ergänzen [23].

Des Weiteren belegen die vorliegenden Daten eine relevante Einschränkung der alltäglichen Tätigkeiten, des Selbstwertgefühls und der Lebensqualität bei annähernd der Hälfte der betroffenen Patienten. Dies deckt sich mit früheren Arbeiten zur Lebensqualität, beruflichen Belastung und Inanspruchnahme von Psychotherapie bei EO [21, 24, 25, 29].

Im Grundrecht ist eine gleich gute Qualität der Gesundheitsversorgung für alle Bürgerinnen und Bürger in Deutschland verankert [14]. Dennoch kann es regionale Defizite in der Versorgung und v. a. im Zugang und in der Erreichbarkeit geben [5]. Bislang fehlten Untersuchungen zur Versorgungssituation von Patienten mit EO in Deutschland. Die vorliegende Betrachtung des Einzugsgebiets des interdisziplinären Mainzer Orbitazentrums ergab, dass die Mehrheit der Patienten aus einem anderen Bundesland als Rheinland-Pfalz stammt und beinahe jeder sechste Patient eine Strecke von 100 km oder weiter in Kauf nimmt. Das Einzugsgebiet für die interdisziplinäre Orbitasprechstunde an der Universitätsmedizin Mainz ist damit sehr groß und könnte ein Hinweis auf Defizite in der Erreichbarkeit für einige Patienten mit EO sein.

Da es sich bei der EO um ein seltenes Krankheitsbild handelt [15], gestaltet sich die Erlangung einer ausreichenden Expertise für niedergelassene Fachärzte schwierig. Die interdisziplinäre Zusammenarbeit an Schwerpunktzentren erlaubt eine sichere Diagnosestellung sowie eine individuell angepasste Therapieplanung und -durchführung. Des Weiteren bieten interdisziplinäre Schwerpunktzentren eine ideale Weiterbildungs- und Forschungsmöglichkeit [31].

In 2019 erfolgt aktuell die dritte Version (PREGO, presentation of Graves’ orbitopathy, III) einer multizentrischen Studie zum Profil der Patienten, die sich erstmalig an einem interdisziplinären Orbitazentrum vorstellen. Die vorherige PREGO-Studie verglich die klinischen Daten von Patienten, die sich in den Jahren 2000 (initiale Studie) und 2012 (zweite Version) erstmalig vorstellten [16]. Es konnte gezeigt werden, dass sich die Zeitspanne zwischen Manifestation der Erkrankung und Vorstellung an den Orbitazentren im Durchschnitt verkürzt hatte. Die PREGO III-Studie wird die vorliegenden Daten sinnvoll ergänzen. Dies ist v. a. aufgrund folgender Limitationen erforderlich: Es handelte sich in der vorliegenden Studie um die retrospektive Aufarbeitung von Querschnittdaten. Es wurden Daten aufgrund bestimmter Einschlusskriterien selektiert, um einen möglichst vollständigen Datensatz zu erhalten. Ein Selektionsbias ist insbesondere dadurch gegeben, dass nur Patienten eingeschlossen wurden, die sowohl in der Endokrinologie als auch in der Augenklinik und im interdisziplinären Board betreut wurden. Außerdem wurden die Daten einer Vorstellung ausgewertet. Informationen zum Verlauf fehlen. Des Weiteren wurden sowohl Erstvorstellungen als auch Daten von Patienten, die bereits länger betreut wurden, analysiert. Gerade aufgrund der letztgenannten Einschränkung, werden die Daten aus PREGO III zusätzliche relevante Informationen liefern. Zukünftige Studien sollten optimalerweise auch beantworten, inwiefern die Vorstellung an einem interdisziplinären Zentrum dazu beiträgt, Behandlungswege von Patienten zu adjustieren.

## Fazit für die Praxis

Interdisziplinäre Orbitazentren versorgen ein breites Spektrum von Patienten mit endokriner Orbitopathie (EO). Insbesondere Patienten mit schwerer Verlaufsform, mit weiteren Autoimmunerkrankungen und bereits vorbehandelte Patienten nehmen weite Anfahrtswege in Kauf. Besonders anspruchsvoll erscheint die Versorgung von Männern mit EO – trotz schwerem Befund fehlt es in dieser Patientengruppe möglicherweise mehr an Eigeninitiative und Krankheitsbewusstsein. Als primärer Ansprechpartner stellt der behandelnde Facharzt eine Schlüsselfigur dar. Eine wichtige Aufgabe von Orbitazentren stellen daher der informative Austausch und die enge Kooperation mit den niedergelassenen Kollegen dar.
